# Combination of Interaural Level and Time Difference in Azimuthal Sound Localization in Owls

**DOI:** 10.1523/ENEURO.0238-17.2017

**Published:** 2017-12-14

**Authors:** Lutz Kettler, Hannah Griebel, Roland Ferger, Hermann Wagner

**Affiliations:** 1 Department of Zoology and Animal Physiology, Aachen University, Aachen D-52074, Germany; 2 Department of Biology, University of Maryland, College Park, MD 20742

**Keywords:** barn owl, cue combination, head-related transfer function, interaural level difference, interaural time difference, sound localization

## Abstract

A function of the auditory system is to accurately determine the location of a sound source. The main cues for sound location are interaural time (ITD) and level (ILD) differences. Humans use both ITD and ILD to determine the azimuth. Thus far, the conception of sound localization in barn owls was that their facial ruff and asymmetrical ears generate a two-dimensional grid of ITD for azimuth and ILD for elevation. We show that barn owls also use ILD for azimuthal sound localization when ITDs are ambiguous. For high-frequency narrowband sounds, midbrain neurons can signal multiple locations, leading to the perception of an auditory illusion called a phantom source. Owls respond to such an illusory percept by orienting toward it instead of the true source. Acoustical measurements close to the eardrum reveal a small ILD component that changes with azimuth, suggesting that ITD and ILD information could be combined to eliminate the illusion. Our behavioral data confirm that perception was robust against ambiguities if ITD and ILD information was combined. Electrophysiological recordings of ILD sensitivity in the owl’s midbrain support the behavioral findings indicating that rival brain hemispheres drive the decision to orient to either true or phantom sources. Thus, the basis for disambiguation, and reliable detection of sound source azimuth, relies on similar cues across species as similar response to combinations of ILD and narrowband ITD has been observed in humans.

## Significance Statement

Owls have evolved a high-performance sound localization system that served as a model for sound localization for >40 years, and is also used as a model for biomimetic applications. The conception so far was, that the owl uses a two-dimensional grid of interaural cues created by its facial ruff and asymmetric ears with interaural time difference (ITD) for localization in azimuth and level differences in elevation. Our study extends this model by showing a major contribution of level differences to azimuthal sound localization in disambiguating unreliable timing difference information. Although auditory processing in barn owls differs from humans, our data reveal remarkable similarities in the mechanism underlying localization and cue combination across species.

## Introduction

Cue combination is a well-known phenomenon in many sensory systems, and has been extensively studied in the visual system (Trommerhäuser et al., 2011). Audiovisual integration improves both visual and auditory perception (Stein et al., 1996; Sekuler et al., 1997; Shams et al., 2000; Arnold et al., 2010; Kim et al., 2015). The combination of interaural time difference (ITD) and interaural level difference (ILD) in human sound localization, known as duplex theory (Strutt, 1907), extends the frequency range over which azimuthal locations may be determined (Buell and Hafter, 1991). In the barn owl, ITDs and ILDs form a two-dimensional grid in which the ITD varies almost exclusively with azimuth, while the ILD varies mainly with elevation, but to some degree also with azimuth (Keller et al., 1998; von Campenhausen and Wagner, 2006). We show here that variation of ILDs in the equatorial plane contributes to the mainly ITD-driven azimuthal localization of a sound source.

ITDs are coded in the brain by a mechanism resembling cross-correlation in neurons that receive precisely phase-locked input from both ears (medial superior olive in mammals, Colburn et al., 1990; nucleus laminaris in birds, Fischer et al., 2008). However, this process is susceptible to ambiguity of the interaural phase difference (IPD) between the signals at the two ears (Stern et al., 1988). The neurons computing ITD only operate in narrow frequency bands and thus cannot distinguish whether the sound at one ear is leading or lagging. This results in cyclical tuning to ITD (Goldberg and Brown, 1969; Carr and Köppl, 2004). In other words, these neurons not only respond maximally to their preferred ‘true’ ITD, corresponding to the location of the sound source, but also to ITDs corresponding to slip-cycle IPDs, thus creating phantom sources (Saberi et al., 1998a,b). To illustrate the occurrence of ambiguities, consider a 5-kHz tone (200-μs period) with an ITD of 100 μs. Since in the barn owl 1° in azimuth corresponds to 2.8 μs (von Campenhausen and Wagner, 2006), the true source in this case would be perceived at an azimuth of +35°, while phantom sources would occur at -35° and +105°. Indeed, narrowband neurons in early stages of the auditory pathway cannot signal the location of the true source unequivocally, but respond to multiple locations (Bremen et al., 2007; Fischer et al., 2008). The phantom sound sources may be regarded as sensory illusions that are perceived in addition to or rivaling the true source. Since natural stimuli, like vocalizations calls of barn owls, may have a very narrow (instantaneous) bandwidth (Bühler and Epple, 1980), the owl should be able to eliminate ambiguity to avoid detrimental behavior. Saberi et al. (1999) reported that a stimulus bandwidth >3000 Hz was needed to entirely eliminate phantom localization. Indeed, remodeling of the representation of ITD (Konishi, 2003; Vonderschen and Wagner, 2014) in downstream nuclei like the external nucleus of the inferior colliculus (ICX) and the optic tectum (OT) creates wideband neurons by across-frequency integration and thus reduces phase ambiguity by suppression of multiple response peaks in neural tuning (Takahashi and Konishi, 1986; Saberi et al., 1999). But what happens if the stimuli are too narrow in bandwidth to allow for unambiguous localization? The stimuli in the study of Saberi et al. (1999) contained a constant ILD of 0dB while natural stimuli may contain varying ILDs. We therefore used naturally occurring variations of ILD in azimuth, revealed by acoustical measurements of the owl head, to test whether small ILDs could help to disambiguate ITD information. Our behavioral experiments using tractable phantom locations demonstrate that these small variations of ILD are sufficient to reduce ambiguous ITD information and thus provide a way to enhance sound localization. These findings extend the model of a two-dimensional grid of ITDs and ILDs in the barn owl by revealing significant influence of ILD on azimuthal sound source localization.

## Materials and Methods

### Care and treatment of animals

North American barn owls (*Tyto furcata pratincola*) were used in this study. The animals were treated and cared for in accordance to the guidelines of animal experimentation and with permission of the Landespräsidium für Natur, Umwelt- und Verbraucherschutz Nordrhein-Westfalen, Recklinghausen, Germany and complied with the NIH Guide for the use and care of laboratory animals. The animals were housed individually or paired under natural light/dark cycle. Before the measurements, a small aluminum bar was implanted on the skull of the owls under anesthesia (anesthesia described below). The aluminum bar allowed for head fixation of the anesthetized animals during head-related transfer function (HRTF)-measurements and for fixation of sensors during behavioral experiments, as well as the earphone frame.

### HRTF measurements

HRTFs were measured in an anechoic chamber (A403, Industrial Acoustics Company GmbH) as described elsewhere (Keller et al., 1998; von Campenhausen and Wagner, 2006). In brief, probe microphones (Sennheiser KE4 211-2) were inserted in the ear canals. The head-related impulse responses (HRIRs) were calculated by cross-correlating the probe-microphone recordings with the raw signal (frequency sweeps from 20 Hz to 16 kHz, logarithmically rising, 500 ms in duration, 5 ms rise/fall time, five repetitions). The HRIRs were afterward corrected by the influence of the hardware setup including the microphones. For that, the HRIR and the impulse response of microphones without a head were Fourier transformed. The resulting Fourier transformations of head and setup were then divided by each other and inversely Fourier transformed into time domain to form the corrected HRIR. The HRTFs were obtained by Fourier transformation of the HRIRs.

Individual HRTFs were recorded from 11 adult owls in total. ITDs and ILDs were calculated from the HRTFs. The individual HRTFs of two owls (owl 1 and owl 2) were used in the behavioral experiments. HRTFs from nine additional barn owls were used for the acoustical analysis only. We shall call the measured HRTFs also “native” HRTFs in the following to discriminate them from HRTFs resulting after manipulations, the “manipulated” HRTFs.

### Behavioral experiments

The tests with two female barn owls (owl 1 and owl 2) were conducted in the same acoustic chamber as the HRTF-measurements. During the behavioral experiments, the owl sat on a perch in the center of the chamber with the head free to move and the legs loosely tied to the perch with falconers jesses. The general behavior was monitored with two infrared cameras, one above and one in front of the owl. Food rewards were provided by a mechanical food dispenser. A red light-emitting diode (LED) was placed in front of the owl at 0° azimuth and elevation and 1-m distance. Before the experiments, the animals were trained to fixate the LED for a few seconds. The LED was later used as a starting cue in an experimental trial. During experiments, the owl initialized a trial by fixating the position of the LED within a window of ±7° in azimuth and ±15° in elevation. During fixation, the LED was automatically switched on for 1-2.5 s. After this period, the LED was automatically switched off, and the stimulus was either presented via loudspeakers (Visaton VRS 8) or earphones which were mounted on a custom build frame (Philips SHE2550). Free-field sessions were performed separately from earphone sessions. The earphone frame was not attached during free-field sessions. In this case only the sensor of the head tracking system was attached to the aluminum bar. When hearing the sound, the owl turned its head toward the perceived sound source. The bird was rewarded if it hit a target window (±7° azimuth and ±15° elevation) around the positions of the true or the phantom sources. To keep the owl under stimulus control, care was taken that the daily reward rate did not drop below 70%.

In the first experiment, we investigated phantom source localization with virtual stimuli and free-field stimuli. The virtual stimuli were presented via earphones and had flat frequency spectra. The ITD was randomly varied between trials (±150, ±100, and ±50 μs); the ILD was set to 0 dB. We called this condition “ITD alone” to distinguished it from stimuli that contain natural ILDs and spectral cues. Free-field stimuli were delivered from six positions in space, corresponding to the ITDs in the ITD-alone condition (±52.5°, ±35°, and ±17.5° in azimuth, all at 0° elevation).

In the second experiment, all stimuli were presented via earphones. Differing from the first experiment, the stimuli were additionally filtered with HRTFs and thus contained all natural cues as in free-field stimuli, including different ILDs. Stimulus ILD and ITD were confirmed after generating the HRTF-filtered signals with the microphones still inserted in the ear canal. During experiments the earphone speakers were not sealed. Sound directions corresponded to ±52.5°, ±35°, and ±17.5° in azimuth, all at 0° elevation. Acoustic crosstalk between earphone loudspeakers was below -20 dB. In addition to using the native HRTFs for stimulation, we also took the advantage of HRTFs being filters that may be changed digitally. We, thus, manipulated individual spatial cues. Specifically, we manipulated ILD independently from ITD as described elsewhere (Poganiatz and Wagner, 2001). Briefly, the average power within a specific frequency band was calculated for the individual HRTF of each ear and was then multiplied by the corresponding factor to gain a set of HRTFs with fixed ILDs. This corresponded to an increase of the monaural gain in one ear and a reduction of the monaural gain in the other ear to keep the average binaural level constant. The phase spectrum of the HRTFs was kept unchanged. The HRTF-filtered stimuli were either presented with native ILDs (HRTF_native_), or the ILDs were fixed to 0 dB (manipulated HRTF_0dB_, in brief HRTF_0dB_) or ILD was fixed to an ILD corresponding to the position of the estimated phantom sound source (manipulated HRTF_phantom_, in brief HRTF_phantom_).

In both experiments, noise bursts with different bandwidths and pure tones were used for stimulation. The tonal frequency or the center frequency of the noise was 5000 Hz. A stimulus frequency of 5000 Hz has a period of 200 μs. This corresponds to 70° in azimuth if converted by 2.8 μs/°, the rate of change of ITD with stimulus direction in the frontal space (von Campenhausen and Wagner, 2006; see also our own measurements below). The location of phantom sound sources of narrowband stimuli was estimated from the period of the center frequency and the conversion factor. Since the location of the phantom was shifted by 70° from the true source, and since the largest absolute stimulus angle in the free field was 52.5°, phantom sources were always located in the hemifield contralateral to the true sound source. The noise bandwidth was varied randomly from 250 to 5000 Hz between trials (250, 500, 750, 1000, 1500, 2000, 3000, 4000, and 5000 Hz). We also used broadband noise (8-kHz bandwidth) as a reference. The spectra of the signals were flattened before bandpass filtering by filtering the raw signals with the inverse transfer function of the electronical setup including the loudspeakers. In experiment two, signals were filtered with the HRTF. In this experiment only pure tones, 1500-Hz bandwidth and 8000-Hz bandwidth were tested.

The stimuli were generated by a custom written MATLAB-Program and converted into analog signals with an I/O-Processor (RX8, Tucker Davis Technology, System III). The signals were also amplified (Ecler MPA 4-80 in loudspeaker stimulation and Denon AVR-1905 in earphone stimulation). The average binaural level with earphone stimulation was 15-dB SPL and thus well above the hearing threshold of barn owls. In free-field conditions, the signal-to-noise ratio was 15 dB at the position of the owl’s head. The duration of each stimulus was 50 ms, which included 5-ms linear onset/offset-ramps.

### Data analysis

After hearing a sound, the owl typically responded with a swift saccadic head turn followed by a fixation period. The angle of the owl’s gaze was measured with a head tracking system (Ascension trakSTAR) which was mounted on the same aluminum bar that allowed fixation of the owl’s head during HRTF-measurements. We analyzed the head orientation angle while the owl fixated the sound source. The condition for trial validity was that the maximum turning velocity exceeded 20°/s, and the response latency was between 50 and 1000 ms. Response latency was the delay time between stimulus onset and onset of the saccadic reaction. Latency was defined as the time that elapsed between stimulus onset and the point in time when the head orientation deviated >5° from the orientation at stimulus onset. In valid trials the response started after the whole stimulus duration (50 ms), i.e., the owl was not responding to an ongoing stimulus while turning its head. The fixation angle was determined as the mean orientation angle within the first 80 ms after the head turning velocity dropped again below 20°/s (Kettler and Wagner, 2014). The fixation angle was corrected by the angle of head orientation at stimulus onset if the stimulus was played with earphones. The owls typically turned either toward the true sound source or toward a phantom sound source in the opposite hemifield depending on the stimulus type. We quantified the number of turns toward the true source and phantom source, respectively, using custom written MATLAB (The MathWorks) scripts.

### Electrophysiology

Standard extracellular recordings from ICX neurons were available from other studies (Vonderschen and Wagner, 2009; Singheiser et al., 2012) in addition to basic neuronal characterization data from studies that are in preparation to be published but not further analyzed in these studies. The basic characterization of neurons are a standard procedure performed at the beginning of a recording to identify the neuron type. Data from 29 adult owls of either sex were available for the analysis of the distribution of best ILDs in both brain hemispheres. The procedures of owl handling, surgery, signal generation, and data acquisition have been described elsewhere (Vonderschen and Wagner, 2009; Singheiser et al., 2012). Procedures were approved by the Landespräsidium für Natur, Umwelt- und Verbraucherschutz Nordrhein-Westfalen, Recklinghausen, Germany and complied with the NIH Guide for the use and care of laboratory animals.

Briefly, the owl was food deprived and its health state was assessed the day preceding the experiment. The animal was sedated with an intramuscular injection of diazepam (Valium, 1 mg/kg; Ratiopharm) 30 min before anesthesia was induced. An analgesic (Temgesic, 0.06 mg/kg; Essex Pharma) was also administered. Anesthesia was induced by an intramuscular injection of ketamine (20 mg/kg; Ceva). Atropine (Atropinsulfat, 0.05 mg/kg; Braun) was administered intraperitoneally to prevent salivation. The owl was then covered by a jacket to restrain the animal and reduce the risk of self-induced injuries. Anesthesia was maintained by regular intramuscular injections of valium and ketamine (approximately every 2 h). A small craniotomy was then performed to expose the brain and a tungsten electrode was lowered into the brain. The scalp was closed after recording and the animal was monitored in a recovery box for the next 12-36 h.

Experiments were performed in a similar anechoic chamber as the behavioral experiments. Dichotic broadband noise stimuli were presented to determine tuning properties of a neuron by varying either ITD or ILD and record the response rates. ITD was varied between -330 μs and 330 in 30-μs steps and ILD kept at 0 dB to measure the ITD tuning curve (firing rate vs ITD). ILD tuning curves where measured by varying ILD from -24 to 24 dB in 4-dB steps while keeping the ITD at the value that evoked the maximum response (best ITD). Tuning to ILD was characterized by the ILD that elicited the highest firing rate (best ILD). This best ILDs and best ITDs of 395 single and multi-units were collected. From these units those were further analyzed that had best ITD and best ILD pairs that also occurred on the equatorial plane as measured in the HRTF measurements. Since ILD varied across owls in the equatorial plane, we also included neurons with best ILDs that were ±4 dB smaller or larger than ILDs found in the HRTF measurements at 0° elevation.

### Statistics

Statistical analysis was performed by using the MATLAB statistics toolbox. For behavioral experiments, the percentage of true-source fixations between the different stimulus types was compared by using contingency tables and tested with a Pearson’s χ^2^ test. Significance between preferred ILDs of neurons of the left and right hemisphere was tested with a two-sided *t* test (Matlab function ttest2).

## Results

### Acoustical measurements

Natural sounds arriving at the ears are composed of multiple spatial cues. ITD, ILD, and monaural spectral cues carry information about the direction of a sound source (Blauert, 1996). In owls, ILD is the major cue for elevational sound localization (Poganiatz and Wagner, 2001) and, thus far, has not been shown to influence detection of the azimuth of a sound source in this species. However, previously published data indicate that ILD also varies in the equatorial plane (see Fig. 5 in von Campenhausen and Wagner, 2006). To examine a possible influence of ILD in azimuth we determined the distribution of ILDs and ITDs from HRTFs. The HRTF characterizes the influence of the body, the head and the outer ear on the sound originating from a point in space. It contains the whole spatial information available in a free-field stimulus. Virtual auditory stimuli may be created by filtering a signal with a pair of HRTFs recorded at the left and right ears, and presenting these via earphones. This mathematical operation also allows for manipulation of the spatial cues, and for creation of stimuli that would never occur in nature but that are helpful for examining the mechanisms used for sound localization.

**Figure 1.**
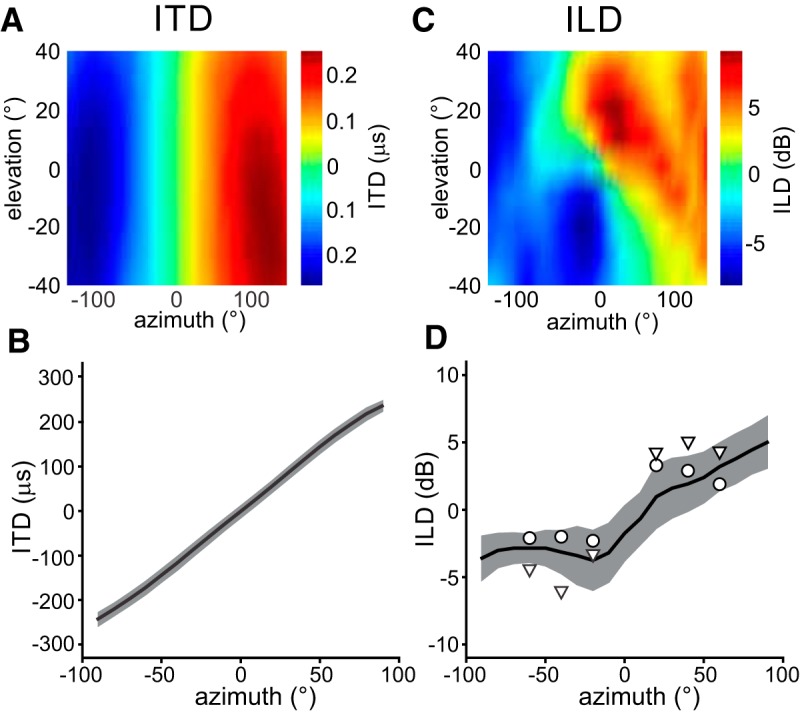
Acoustical measurements of the owl head. ***A***, Surface plot illustrating distribution of ITD in the frontal space. The data are derived from HRTF measurements of one owl which was not used in the behavioral experiments. ***B***, Average ITD of nine owls at 0° elevation (black line). ***C***, Surface plot illustrating distribution of ITD in the frontal space for the same owl as in ***A***. ***D***, Average ILDs of the same nine owls in the equatorial plane (0° elevation) with broadband stimuli. Gray areas show SD. ILDs of the two owls used in the behavioral experiments are shown with circles (owl 1) and triangles (owl 2).

The acoustical measurements showed that ITD varies almost exclusively with azimuth as illustrated by the example for one owl in Figure 1*A*. HRTF recordings of nine owls show that ITDs occurring in the frontal space of the barn owl vary almost linearly between ± 250 μs at ±100° azimuth with low variability, corresponding to a physiological ITD range of 500 μs (Fig. 1*B*). Poganiatz et al. (2001) identified ITD as the major cue for azimuthal localization, which is consistent with the observation that ITD is mostly invariant to elevational changes. The color gradient in Figure 1*C* illustrates that ILD also varies with azimuth, but to a lesser extent as with elevation. The HRTFs of nine owls demonstrated a clear dependence of ILD on azimuth in the equatorial plane with negative ILDs occurring in the left hemifield and positive ILDs occurring in the right hemifield (Fig. 1*D*). The azimuthal dependence of ILD was not symmetric around 0° azimuth, but had a complex shape. The range of ILDs in the equatorial plane (13.8 dB) was small compared to the overall ILD range (40 dB; von Campenhausen and Wagner, 2006). As a recent human study revealed (Hartmann et al., 2016), such small ILDs may be sufficient to influence azimuthal localization in that they enhance the localization of the azimuth. In the study of Hartmann et al. (2016), manipulating the ILD by using virtual stimuli increased or decreased laterality of the perceived stimulus. A similar effect might be found in owls, since they generally use ITD and ILD in the same frequency. For that reason, we performed behavioral experiments with free-field and virtual stimuli to test this hypothesis.

### Behavioral experiments

Two experiments were conducted with two barn owls to test the influence of ILD on azimuthal sound localization. Data from 11,056 behavioral trials were collected. Each data point is based on at least 20 trials. In experiment 1a, dichotic signals were presented with earphones and the ITD was varied between trials (owl 1: 1970 trials; owl 2: 1856 trials). The owl had to localize flat spectrum noise bursts with different bandwidths. In experiment 1b (owl 1: 2242 trials; owl 2: 1778 trials), the same bandpass noise signals as in experiment 1a were played from free-field loudspeakers at the locations corresponding to the ITDs in experiment 1a and 0° elevation. Note that free-field stimuli contain all natural cues, including location-dependent ILDs, while in the stimuli used in experiment 1a, the ILD was set to 0 dB. In experiment 2, the owls also had to respond to noise with different bandwidths. The stimuli were presented with earphones, but were filtered with HRTFs corresponding to the speaker positions in experiment 1. We used native HRTFs which contain all natural cues and are equivalent to free-field stimulation as in experiment 1b (HRTF_native_; owl 1: 828 trials; owl 2: 550 trials). We also manipulated the ILD but left all other cues as they were in the native HRTFs. The ILD was either fixed to 0 dB, which corresponds to the parameters used in experiment 1a (HRTF_0dB_; owl 1: 445 trials; owl 2: 513 trials), or we fixed the ILD to the value measured 70° off the true sound source at the estimated position of the phantom source in the contralateral hemisphere (HRTF_phantom_; owl 1: 420 trials; owl 2: 454 trials).

### Characteristics of head turns

**Figure 2.**
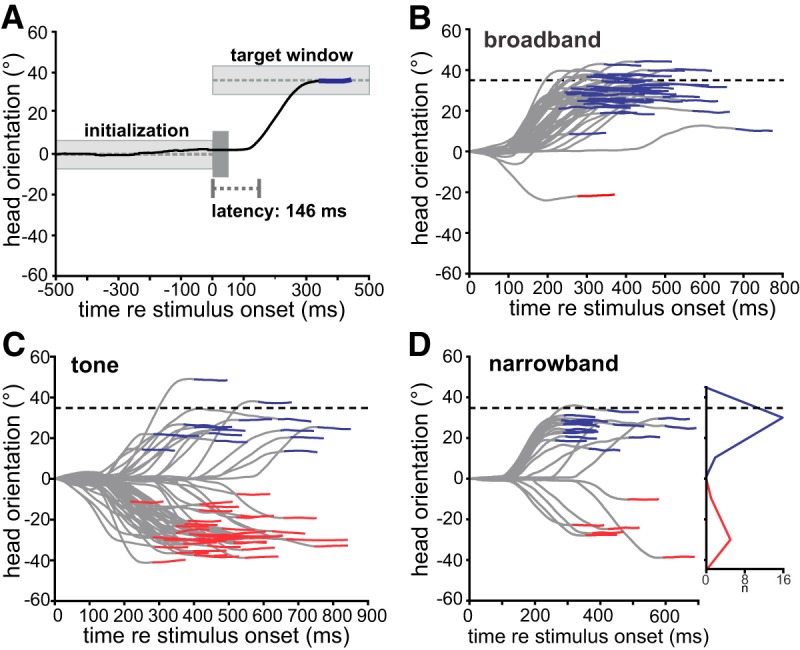
Localization behavior in ITD-alone condition and head turn analysis. The owls localized dichotic noise signals with a center frequency of 5 kHz. The bandwidth was varied between trials. The directional information was provided by ITDs; ILD was set to 0 dB. Data shown for owl 1. ***A***, Head orientation for a single trial with broadband noise and 100-μs ITD corresponding to 35°. The owl was trained to direct its gaze toward a virtual starting window at 0° azimuth and elevation to initiate a stimulus trial. After keeping this orientation for some 1-2.5 s (gray area initialization), the stimulus was played. The owl typically responded with a head saccade after a short latency followed by fixation at the sound source. Negative and positive angles denote turns to the left and right. Abscissa shows time with respect to stimulus onset at 0 ms. ***B***, All head turns with 100-μs ITD and broadband noise (center frequency = 5 kHz; bandwidth = 8 kHz). Blue lines indicate fixations at the true sound source. Fixations in the opposite hemifield were classified as phantom fixations (red lines). ***C***, All head turns with 5-kHz pure tones and ITD = 100 μs. ***D***, Responses to narrowband noise (bandwidth = 1.5 kHz). The inset in ***D*** shows the distribution of fixations with narrowband noise.

When hearing a faint sound, barn owls naturally turn their head toward the perceived position of the sound source. Figure 2*A* shows the time course of head orientation in a single trial. After the owl oriented its gaze to the front within the boundaries of the initialization window (see Materials and Methods) for some time, the stimulus was played. In this example, a broadband noise burst with an ITD of 100 μs was played. Figure 2 shows data from owl 1 as example. The owl responded to the sound after a latency of 146 ms with a saccade toward the true virtual sound source at 35°. The head orientation after the saccade was held within the target window for a sufficient time, and the owl was rewarded after a predetermined short time (see Materials and Methods). The owl almost always (>90%) turned to the right, i.e., the true source, with 100-μs ITD and broadband noise as illustrated in Figure 2*B*. By contrast, when the owl was stimulated with a 5-kHz tone with an ITD of 100 μs, the owl turned more often to the hemifield opposite of the true source (Fig. 2*C*). In the following turns toward the hemifield opposite to the true source are termed phantom source fixations. More specifically, during phantom source fixations the owl turned its head to a position roughly off by one stimulus period (70° at a frequency of 5000 Hz and a period of 200 μs) from the true source. The owls generally tended to undershoot both the true as well as the phantom source; a commonly observed behavior in owls (Hausmann et al., 2009; Kettler and Wagner, 2014). The percentage of phantom-source fixations was intermediate with a narrowband stimulus (Fig. 2*D*, center frequency 5000 Hz, bandwidth 1500 Hz). Fixation angles in all cases were bimodally distributed with a null at 0° (Fig. 2*D*, inset) which made it easy to link fixations to the true or the phantom sources and to calculate percentage of true source fixations per bandwidth and stimulus condition.

### Experiment 1. Influence of bandwidth on phantom-source fixations

**Figure 3.**
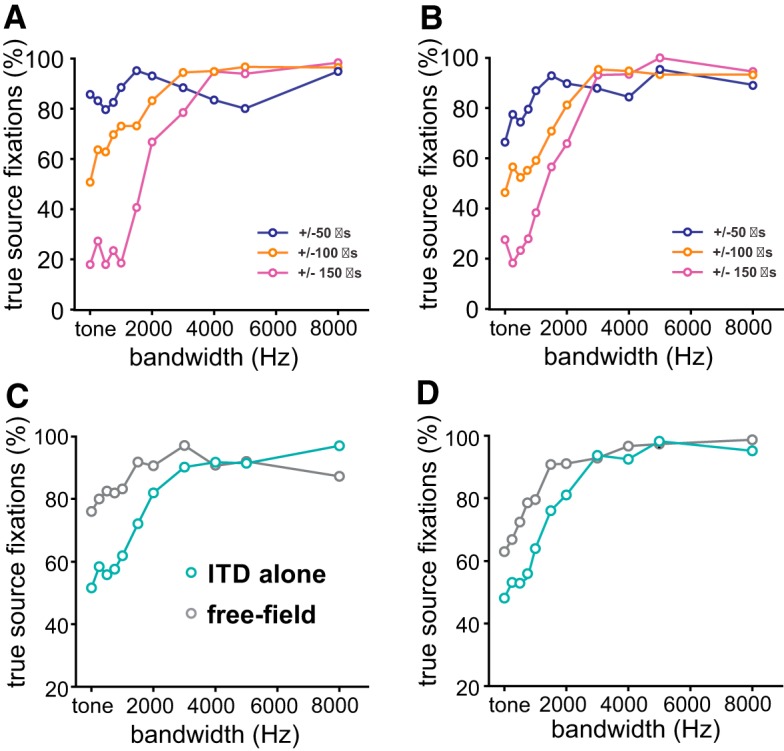
Experiment 1. Phantom source localization with bandpass-filtered dichotic signals and free-field signals. Percentage of true source fixations as a function of stimulus bandwidth (0 Hz: pure tone stimulation) for owl 1 (***A***, ***C***) and owl 2 (***B***, ***D***). ***A***, ***B***, Average of the fixations at mirrored ITDs indicated by the inset. ***C***, ***D***, Average across all stimulus directions for dichotic stimuli with 0-dB ILD (ITD alone, cyan) and free-field (gray) stimuli. The percentage of phantom source fixations is equal to 100% minus the percentage of the turns toward the true source.

Since perceiving and responding to phantom-source illusions may create detrimental situations in natural behavior, we hypothesized that the owl should have the ability to reduce errors for narrowband stimuli. We first confirmed and quantified an earlier observation by Saberi et al. (1999) that phantom source localizations depended on stimulus bandwidth: turns toward the true source in ITD-alone condition increased with increasing bandwidth up to 90% above 3-kHz bandwidth in both owls (Fig. 3). This conclusion was drawn after averaging the responses to mirrored ITDs (Fig. 3*A*, owl 1, *B*, owl 2) which also effectively eliminated a bias for left sources from the data observed in both owls (Fig. 2*C*). Large ITDs (±150 μs) also elicited fewer turns toward the true source than small ITDs (±50 μs; Fig. 3*A*,*B*). Thus, sound sources closer to the center of the gaze, both the true source and the phantom source, dominated over peripheral sound sources. This behavior is consistent with the owls’ preference for frontal locations (Hausmann et al., 2009; Fischer and Peña, 2011; Kettler and Wagner, 2014).

In the next step, we compared the results of stimulus presentation via earphones (ITD-alone stimulus) and in free field (Fig. 3*C*,*D*). For both earphone presentation and free-field stimulation, the percentage of true-source fixations increased in both owls as the bandwidth of the stimulus increased (Fig. 3*C*,*D*). In other words, the average percentage of phantom-source fixations over all six directions decreased as the bandwidth of the stimulus increased. However, the dependence on bandwidth differed between stimulus paradigms. The difference is largest for tonal stimuli. While tonal stimuli in ITD-alone signals elicited 50% turns to either source, free-field tonal stimuli resulted in more turns to the true source (Fig. 3*C*,*D*). The difference was statistically significant in both owls. For the statistical test, the data were averaged across stimulus position (contingency tables, Pearson’s χ^2^ test, *p* < 0.001 in both owls). The free-field psychometric curves were shifted to the left compared with the ITD-alone psychometric curves up, indicating better performance in the former paradigm compared with the latter paradigm. For example, 80% true-source fixations with free-field sounds was already reached at bandwidths of 250 Hz (owl 1) or 1000 Hz (owl 2) in the free-field paradigm, while a bandwidth of 2000 Hz was necessary in both owls to reach a performance of 80% performance in the ITD-alone paradigm (Fig. 3*C*,*D*). In other words, the addition of spatial cues as present in free-field stimuli partly compensated for ambiguities and helped to eliminate the illusion. Referring to the small ILDs occurring in the equatorial plane as revealed by the acoustical measurements (Fig. 1), we hypothesized that this additional information may be carried by ILDs.

### Experiment 2. Influence of ILD on azimuthal localization

**Figure 4.**
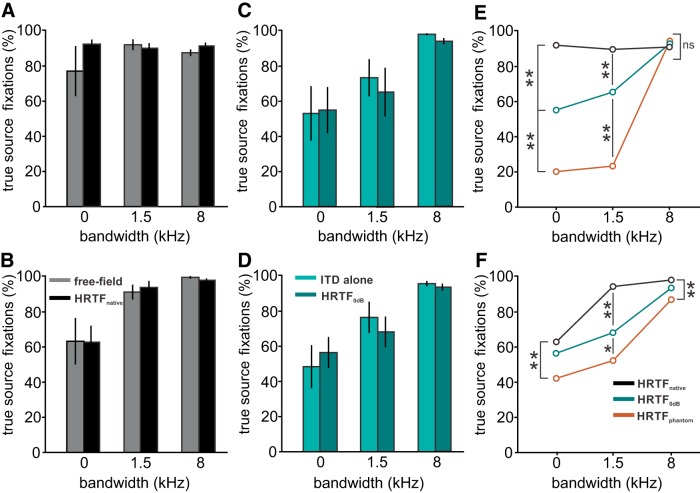
Experiment 2. Behavioral experiments with HRTF-filtered stimuli. Upper row, data from owl 1. Lower row, data from owl 2. The plots show the average percentage of turns toward the true source across all stimulus directions in response to noise with different bandwidth (1.5 kHz, 8 kHz) and tones (0 kHz). ***A***, ***B***, Comparison between free-field experiments (gray) and experiments with native HRTFs (black). ***C***, ***D***, Earphone stimuli with flat spectra and ILD = 0 dB (ITD alone, light-cyan) and HRTF with ILD fixed to 0 dB (HRTF_0dB_, dark cyan). ***E***, ***F***, HRTF-filtered stimuli with the ILD fixed to the value measured at the direction of the phantom sound source. Error bars show SEM over six stimulus locations used in this study (±52.5°/±150 µs, ±35°/±100 µs, and ±17.5°/±50 µs); **p < 0.01, *p < 0.05 with Pearson’s χ^2^ test; ns, not significant.

In the second experiment, we tested the hypothesis that ILD may improve true-source localization. To determine the influence of ILD we used native and manipulated HRTFs as stimuli. We first checked whether the native HRTFs indeed contained the azimuthal information of the free-field stimuli as is theoretically expected (HRTF_native_) by comparing the head-turning behavior to free-field stimulation. The tests were conducted with a 5-kHz tone, narrowband noise centered at 5 kHz with a bandwidth of 1.5 kHz and broadband noise, also centered at 5 kHz but with a bandwidth of 8 kHz. No statistical difference was found between stimulation with native HRTFs and free-field stimulation in either owl for five of the six stimulus conditions (contingency tables, Pearson’s χ^2^ test; Fig. 4*A*,*B*). Only data in owl 1 when a tone was used a stimulus yielded a difference with a higher percentage of true-source fixations with native HRTFs than with free-field stimuli. This data, including the data point where a difference occurred, demonstrated that the stimulation with HRTF_native_ is equivalent to stimulation with free-field sources by eliciting a better performance than ITD-alone stimuli.

In the next step, we manipulated the ILD of the HRTF-filtered stimuli. We first set the ILD to 0 dB (HRTF_0dB_). This created a situation similar to the ITD-alone condition in experiment 1 except that the stimuli contained a natural frequency spectrum. The behavior of both owls with HRTF_0dB_ nevertheless closely resembled their behavior with ITD-alone stimuli. Specifically, phantom-source localization percentages were not statistically different (Fig. 4*C*,*D*; contingency tables, Pearson’s χ^2^ test, *p* > 0.05). Since ILDs were 0 dB in both experiments and thus pointing toward 0° azimuth and elevation this result suggested an influence of ILDs in phantom-source fixations.

**Figure 5.**
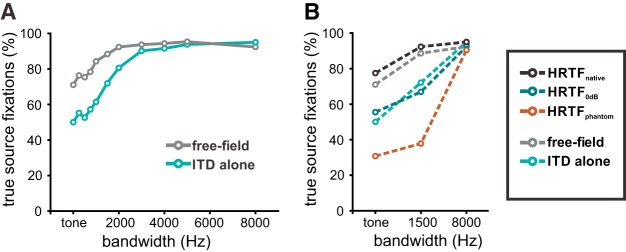
Average behavior of two owls and summary of the behavioral data. ***A***, Average true source fixations over all stimulus directions for free-field (black) and ITD-alone conditions (cyan). ***B***, Comparison of experiment 1 (free field, ITD alone) and experiment 2 (HRTF).

In the last test, ILDs were fixed to the ILD measured at the phantom source (HRTF_phantom_) to substantiate the conclusions drawn from the previous experiments. The expectation was that phantom source fixations should increase if the ILD was used by the owls to compute the position of the sound source. This effect should be most pronounced for tonal stimulation, because in these stimuli, ITD information is maximally ambiguous. Tonal true-source fixations indeed decreased significantly compared to HRTF_native_ stimulation in both owls (Fig. 4*E*,*F*; contingency tables, Pearson’s χ^2^ test, *p* < 0.01 in both owls). The reduction was still present in both owls for a stimulus with a bandwidth of 1.5 kHz (Fig. 4*E*,*F*). It disappeared in owl 1 for the broadband stimulus (Fig. 4*E*), but not in owl 2 (Fig. 4*F*). This indicated a diminishing weight of ILD on azimuthal sound source localization with increasing bandwidth. However, and more importantly, this finding also shows that ILDs indeed influence azimuthal sound localization in barn owls. Figure 5 provides a summary of the aforementioned effects by showing the average performances of the two owls. Free-field sources are less ambiguous than dichotic stimuli with an ILD pinned to 0 dB (Fig. 5*A*). If the additional ILD in native HRTFs matches the direction of the ITD, the performance is very similar to the performance with free-field stimulation. Setting the ITD to 0 dB in the ITD-alone and HRTF_0dB_ paradigms, leads to similar responses, independently of the presence of detailed spectral cues. The behavioral performance is reversed if the ILD in the stimulus points toward the phantom source. In other words, true source fixations in the HRTF_phantom_ case roughly correspond to phantom-source fixations in the HRTF_native_ paradigm (Fig. 5*B*).

### Quantifying ILD representation in ICX

**Figure 6.**
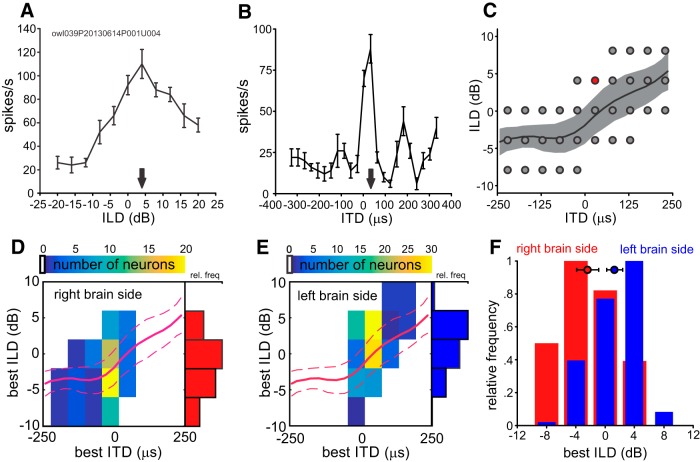
Representation of azimuthal ITD and ILD in the external nucleus of the inferior colliculus (ICX). ***A***, Typical ILD tuning curve as measured in left ICX. Response rate is plotted as a function of ILD. The ILD that evokes the highest response rate (best ILD, +4 dB) is shown with a black arrow. ***B***, ITD tuning curve of the same neuron as in ***A***. Response rate is plotted as a function of ITD. Black arrow indicates best ITD (+30 μs). ***C***, Indicated by the black line are the ILD/ITD combinations occurring on the equatorial plane. Shaded area shows SD of ILD. The circles indicate the best ITD/best ILD pairs from the total population of recorded ICX neurons considered in the following analysis. Each circle depicts the center of a histogram bin, which includes all best ITD/ILD of corresponding values. The value for the neuron in ***A***, ***B*** is shown as a red filled circle. ***D***, ***E*,** Distributions of best ITDs and best ILDs in the left and right ICX. Red line shows the ITDs and ILDs occurring on the equatorial plane as in ***C***. Placed underneath are the histogram data of best ILDs and ITDs (from electrophysiological recordings). The color code shows the number of units with preferred ITD/ILD pairs in a histogram bin. Bin width for ILD is 4 dB, and 60 μs for ITD. More negative ILDs were found in the right hemisphere, while more positive ILDs were found in the left ICX. Data basis: 395 single neurons or multi-units from ICX. Distribution is shown for those neurons that match the dots in ***C*** (left: *n* = 76, right: *n* = 109). ***F***, Distribution of best ILDs in the ICX in the left (blue) and right (red) brain side summarizing data from ***D***, ***E***. Color filled circle indicate mean best ILD for left (blue) and right (red) brain side. Error bars show 99.9% confidence intervals. Negative ITD and ILD values refer to left ear earlier or left ear louder.

One possible explanation for the behavioral effects of ILD might be an asymmetry of ILD representation in the two brain hemispheres. Rival hemispheres could drive the behavioral decision to turn to either phantom or true sound sources. It has been shown that in the OT each hemisphere represents the contralateral auditory space (Knudsen, 1982), such that negative ITDs dominate in the right hemisphere and vice versa (Olsen et al., 1989). Olsen et al.(1989) also found a gradient of ILD representation perpendicular to the ITD representation, but their data did not reveal how ILD is represented in the equatorial plane. In the ICX, spatial receptive fields are generated by convergence of ITD and ILD sensitive pathways. Thus, ILDs on the equatorial plane may be also asymmetrically represented in the midbrain hemispheres. Our data confirm this assumption (Fig. 6). The examples in Figure 6*A*,*B*show a typical ILD tuning curve and an ITD tuning curve of a single neuron. The largest response rate (best ILD) of this neuron was at -4 dB and the best ITD at 60 μs. Best ILD and ITD were used as signature for the preferred direction of a neuron. From a population of 395 ICX neurons, we analyzed those 185 with best ITD and best ILD (Fig. 6*C*, gray dots) that matched combinations of ITD and ILD on the equatorial plane in the HRTF recordings (Fig. 6*C*, black line average of nine owls). We included neurons with best ITD/ILD combinations near the average values from the HRTFs of nine owls. Best ILDs and ITDs were analyzed in bins with a width of 4 dB and 60 μs (Fig. 6*C*, dots). For example, the neuron in Figure 6*A*,*B* lies in the bin centered at +4-dB ILD and +45-μs ITD as indicate by the red dot in Figure 6*C*.

The data show that neurons in the right external nucleus of the ICX respond preferably to negative ITDs (left ear leading) and negative ILDs (left ear louder), while neurons in the left ICX respond preferably to positive ITDs and positive ILDs (Fig. 6*D*,*E*). We observed a mean best ILD of 1.28 dB for units of the left hemisphere and -2.37 dB for units of right hemisphere (Fig. 6*F*, two-sample unpaired *t* test, *p* < 0.001, *t* = 6.8861, degrees of freedom = 183).

## Discussion

We analyzed the contributions of ILD to azimuthal sound localization by combining acoustical measurements, virtual stimulation, psychophysics, and electrophysiology in barn owls. Combining ITD and ILD information can eliminate an auditory illusion, namely the phantom sound sources that occur with narrow band stimuli. Our data show for the first time that the owl does not exclusively use ILDs for elevational sound localization, but also for azimuth. This use of ITD and ILD cues in barn owl and human sound localization provides an auditory example of cue combination.

The standard model for barn owl sound localization states that ITD and ILDs code for different spatial coordinates (Moiseff, 1989; Poganiatz and Wagner, 2001; Poganiatz et al., 2001). ITDs varied almost exclusively with azimuth in the whole physiological frequency range of the owl (Fig. 1; Keller et al., 1998; von Campenhausen and Wagner, 2006). Experiments demonstrated that ITD is the main cue for azimuthal sound localization (Moiseff, 1989; Poganiatz et al., 2001; Hausmann et al., 2009). Poganiatz et al. (2001) demonstrated that, even if ILD is pointing at a different location than ITD, the owl turned its head toward the location determined by the ITD, indicating that ITD is the dominant cue in broadband signals. However, not all observations, such as front-to-back confusion in azimuthal sound localization (Hausmann et al., 2009), could be sufficiently explained by the standard model. Thus, it seemed that other cues must also influence azimuthal sound localization. We identified ILD as disambiguating cue when the ITD provides only ambiguous information. ILDs are appropriate cues, since they vary with azimuth in the equatorial plane. We show here that the small variations of ILD in azimuth present in the signal at the ear drums suffice to enhance localization.

Saberi et al. (1999) speculated that owls perceive two images in case of ambiguous signals and choose one. The images are assumed to be generated by the tuning periodicity of neurons that perform a cross-correlation of narrowband sounds (Saberi et al., 1998b; Fischer et al., 2008). Such a neuron responds maximally to two (or more) directions with tonal stimulation. Here, we show that one stimulus may cause the owls to localize either the true or the phantom sources. Furthermore, the decision of the bird to turn to either of the sources varied from trial to trial. Responses of a single neuron do not allow for prediction of these behaviors, however, since a single neuron in the auditory space map can only represent one spatial direction. Hence, an ensemble of neurons is needed to elicit the observed behavior. We show here that at a population level, ILDs are asymmetrically represented in the left and right brain sides. We propose that this asymmetric representation might be the basis for rival hemispheres driving the observed behavior.

**Figure 7.**
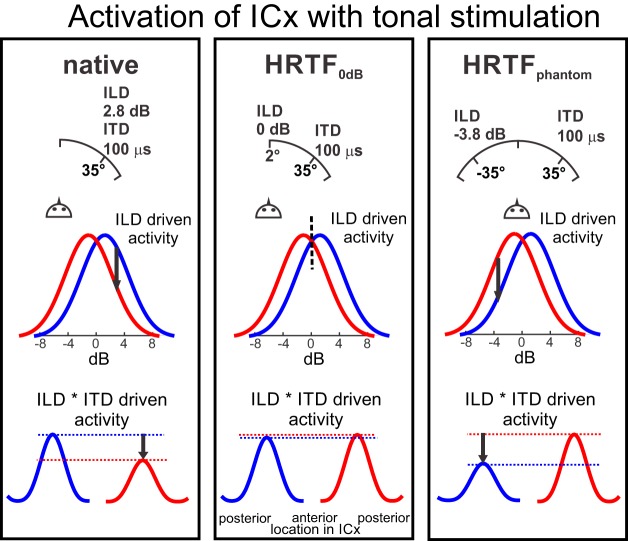
Behavioral data explained by rival brain hemispheres. ILD and ITD form a map of auditory space and interact by multiplication. The figure shows the possible influence of ILD on the ITD-driven activity in both midbrain hemispheres with tonal stimulation as example. Left column, When ILD and ITD point at the same direction, as it is the case with native stimuli, ILD-driven activity is higher in the left brain side corresponding to the true source (blue) than in the right side that corresponds to the phantom source (red). This reflects on the overall activity (lower row). Hence, the owl localizes the true source more often than the phantom source. More frontal ITDs are represented in the anterior ICX, while peripheral ITDs are found in posterior parts of the ICX. Middle column, If the ILD is fixed to 0 dB, the ILD-driven activity in both brain sides is almost equal and, thus, the overall activity, too. The owl then localizes phantom and true sources with the same frequency. Right column, If the ILD is fixed to the value that corresponds to the phantom source, the overall activity is higher in the brain side that corresponds to the phantom source (right brain side for phantoms in the left hemifield). Consequently, the owls turned their head more often to the phantom source.

A simple model that takes the asymmetric representation of space in the two brain hemispheres into account provides an explanation for the observed behavior. The rivalry between phantom and true sound source may reflect competition between brain hemispheres. Figure 7 illustrates the possible contribution of this asymmetry to behavior. Sound sources in the right hemifield evoke greater ILD-driven activity in the left hemisphere than in the right hemisphere. Ambiguous ITD tuning may generate activity in both brain hemispheres reflecting true and phantom source localization. Multiplicative combinations of ITD- and ILD-driven (Peña and Konishi, 2001) activity with native stimuli reduces the activity in the brain hemisphere corresponding to the phantom source. ITD-driven activity is not influenced if ILD-driven activity is equal in both hemispheres (HRTF_0dB_). The activity in the brain hemisphere of the phantom source is increased if ILD is fixed to the position of the phantom source (HRTF_phantom_). The bell-shaped ILD tuning curves in ICX are very broad with an average half-height width of 17 dB (Pérez et al., 2009) and thus carry only poor information within the range of ILDs occurring in the frontal space (13.6 dB). Thus, it is unlikely that the owl could rely on ILD alone to precisely localize the azimuth of a sound sources. It is more likely that ITD is the primary cue in determining the location of the source, and that ILD provides a weighting factor for ambiguous sources.

In contrast to barn owls, it was long known that both ITD and ILD influence azimuthal sound localization in mammals and human. ITD and ILD information is combined across different frequency bands (Buell and Hafter, 1991), with ILD being the main cue in the high-frequency range and ITD being the main cue in the low-frequency range (Strutt, 1907). However, conflicting ITD and ILD information, e.g., in manipulated virtual stimuli, may lead to the perception of a single sound source that lies between the locations determined by ITD and ILD (Young and Carhart, 1974). In other cases two images are perceived (Whitworth and Jeffress, 1961). The weighting of these cues is frequency dependent. For example, broadband ITD information overrides ILD information that points to a different direction than the ITD (Wightman and Kistler, 1992), which is similar to the data reported here and in Poganiatz et al. (2001). The transition of dominance of either cue depends mainly on the head size and the critical frequency at which phase ambiguities may appear. For example, Keating et al. (2014) showed that the situation in ferrets is similar to humans, with a slightly higher critical frequency, but also that the weighting of the different cues can change during development (Keating et al., 2013). Furthermore, a most recent report of human data (Hartmann et al., 2016) showed that even small naturally occurring ILDs at low frequencies eliminate ambiguities that arise from ambiguous IPDs (>180°) with pure tones, while ambiguity was increased by unnatural combinations of ITD and ILD. These observations are congruent with the data reported here, in that ILD helps to disambiguate the source of narrowband stimuli in owls. Apparently, ITDs and ILDs are combined in the same frequency band in humans and owl, which indicates a similar mechanism underlying combination of these cues in both species.

One interesting general question is how cues are combined (for a review, see Ernst, 2006). An additive (linear) interaction means that both cues contribute to the position estimate at any time (Gu et al., 2008). Nonlinear interactions occur, for example, in depth vision where binocular disparity and motion parallax interact nonlinearly in the computation of depth (Bradshaw and Rogers, 1996), contour perception in the visual system (Persike and Meinhardt, 2015), or multi-sensory integration (Sheppard et al., 2013). Such adaptive weighting of cues optimizes the detection and localization of stimuli in an environment of constant fluctuation of incoming sensory information. In the present case a nonlinear interaction of ITD and ILD is likely, since these cues are integrated in a multiplicative fashion on a cellular basis (Peña and Konishi, 2001; Fischer et al., 2007). As is the case with ITD for narrowband high-frequency sounds, often one cue is not reliable throughout stimulus space. In addition to the ambiguities that where shown in the presented experiments, the reliability of IPDs, from which ITDs are computed, varies with frequency and sound source location in barn owls (Cazettes et al., 2014). In such a situation, a second (or third) cue may also be able to support the spatial information, as is the case for the ILD cues used to disambiguate ITDs here. The reliability of ITD was low with small bandwidth but the weight of ILD relative to that of ITD increased in this situation, which improved the detection of the true stimulus position. On the other hand, the weight of ILD was low compared to the weight of ITD in broadband conditions when ITD was a reliable cue.

In summary, one cue may not be reliable throughout the whole stimulus space, and may even produce illusory percepts. We showed, that pure ITD-based azimuthal localization is not sufficient to explain the entire behavioral spectrum. In a natural situation, combinations of multiple cues may support the exceptional sound localization abilities of barn owls. We do not rule out that more cues might help in eliminating illusions. Frequency or amplitude modulations for example may also provide additional compensation from ambiguity in a fluctuating sensory world.

## Synthesis

Reviewing Editor: Leonard Maler, University of Ottawa Decisions are customarily a result of the Reviewing Editor and the peer reviewers coming together and discussing their recommendations until a consensus is reached. When revisions are invited, a fact-based synthesis statement explaining their decision and outlining what is needed to prepare a revision will be listed below. The following reviewer(s) agreed to reveal their identity: Jose Pena, G. Christopher Stecker

Editorial summary

Both reviewers note that all the critical comments of their reviews have been very successfully answered and that the resulting Ms is now an important and novel contribution to the literature on the mechanisms of sound localization. I have included the remaining minor changes required. These will only require editorial oversight. Please make sure that you make the changes listed below including a thorough check of grammar and punctuation. Please highlight (color coded) all the changes corresponding the numbered points of Reviewer 1 as well as the grammatical changes.

Reviewer #1

1. Line 326, correct typo.

2. Figure legend 2a: Indicate that “After keeping this orientation for some 1 to 2.5 seconds” corresponds to the 'Initialization' period shown in the figure and that the gray bar represents the stimulus.

3. Figure 3a: correct typo in y-axis label.

4. Figure legend 3a,b: consider revising to 'mirrored ITDs' instead of mirrored stimulus directions.

5. Figure legend 3c,d: indicate in the figure legend that two types of stimulation are used in this figure: earphones and free field.

6. Figure 4. Correct typo in y-axis labels in all panels.

7. Figure 5. Correct typo in y-axis labels in both panels.

8. Lines 424-426: 'optic tectum' mentioned twice in parenthesis for unclear reason.

9. Figure legend 6. The title of the figure 'Representation of auditory space in the midbrain of the barn owl“ sounds somewhat hyperbolic. The figure shows tuning to ITD and ILD in ICx.

10. Figure 6a,b, show an example that appears to contradict (best ITD +60us/best ILD -4dB) the claim that positive best ITDs correspond with positive best ILDs and vice versa. The blue dot in Figure 6c may not correspond to the neuron shown in a and b.

11. Figure legend 6. Revise 'color code shows the amount of units' to 'color code shows the number of units'

12. Lines 472-473. Incomplete sentence 1. Line 326, correct typo.

2. Figure legend 2a: Indicate that “After keeping this orientation for some 1 to 2.5 seconds” corresponds to the 'Initialization' period shown in the figure and that the gray bar represents the stimulus.

3. Figure 3a: correct typo in y-axis label.

4. Figure legend 3a,b: consider revising to 'mirrored ITDs' instead of mirrored stimulus directions.

5. Figure legend 3c,d: indicate in the figure legend that two types of stimulation are used in this figure: earphones and free field.

6. Figure 4. Correct typo in y-axis labels in all panels.

7. Figure 5. Correct typo in y-axis labels in both panels.

8. Lines 424-426: 'optic tectum' mentioned twice in parenthesis for unclear reason.

9. Figure legend 6. The title of the figure 'Representation of auditory space in the midbrain of the barn owl" sounds some dwhat hyperbolic. The figure shows tuning to ITD and ILD in ICx.

10. Figure 6a,b, show an example that appears to contradict (best ITD +60us/best ILD -4dB) the claim that positive best ITDs correspond with positive best ILDs and vice versa. The blue dot in Figure 6c may not correspond to the neuron shown in a and b.

11. Figure legend 6. Revise 'color code shows the amount of units' to 'color code shows the number of units'

12. Lines 472-473. Incomplete sentence.

Reviewer #2

The manuscript does include a large number of grammatical errors throughout, mainly use of commas and mismatching plural/singular case. Presumably these can be sorted out in the editorial process without me pointing them out one by one.
